# Structured Activity and Free Outdoor Play in Early Childhood Education and Care: An OSRAC-P Observational Study of Physical Activity Intensity and Context

**DOI:** 10.3390/children13040527

**Published:** 2026-04-10

**Authors:** Ivana Nikolić, Snježana Mraković, Marijana Hraski

**Affiliations:** Department of Kinesiology, Faculty of Teacher Education, University of Zagreb, 10000 Zagreb, Croatia; ivana.nikolic@ufzg.hr (I.N.); snjezana.mrakovic@ufzg.hr (S.M.)

**Keywords:** preschool children, outdoor physical activity, early childhood education and care (ECEC), physical activity intensity, activity context, OSRAC-P

## Abstract

**Highlights:**

**What are the main findings?**

• The overall intensity of preschool children’s physical activity does not differ significantly across different organizational forms of outdoor time.
• Activity patterns and contexts differ, particularly in activity types, equipment use, and social organization, indicating that structured activity, free play, and free play with portable equipment are associated with different patterns of behavior.

**What are the implications of the main findings?**

• Organization of outdoor time should focus not only on increasing activity intensity but also on the context of movement, as different formats (structured activity, free play, play with equipment) are associated with different patterns of activity types, equipment use, and social organization.
• Varying and combining different outdoor activity formats, including the periodic introduction or rotation of portable equipment, with flexible educator involvement, can support diverse patterns of movement and interaction within existing preschool routines without requiring additional time or major structural changes.

**Abstract:**

Background: Outdoor time in early childhood education and care (ECEC) settings provides important opportunities for children’s physical activity. Evidence is limited on whether different organizational outdoor conditions influence not only activity intensity but also the contextual characteristics of children’s movement. Methods: An observational study was conducted using the Observational System for Recording Physical Activity in Children—Preschool Version (OSRAC-P). The study was conducted in two public ECEC centers. Not all children were observed across all three conditions due to the field-based design. A total of 7440 observation intervals were analyzed from preschool children across three outdoor conditions (structured educator-led physical activity, outdoor free play, and outdoor free play with additional portable equipment) using a momentary time-sampling protocol (10 s observation + 50 s recording), resulting in one interval per minute. Physical activity intensity, activity type, equipment use, and social context were coded. Contextual differences were analyzed using chi-square tests with standardized residuals, and activity intensity using linear mixed-effects models. Results: No significant differences were found between outdoor conditions in physical activity intensity, sedentary behavior, and moderate-to-vigorous physical activity (all *p* > 0.05). About one-third of the variance in activity intensity was attributable to individual differences between children (ICC ≈ 33%). Differences were observed in contextual characteristics. Structured activity involved more locomotor activities and greater adult involvement, with 49.4% of intervals occurring in groups with an educator present. Free play with portable equipment showed more manipulative activities, greater equipment use, and mostly peer interactions without adult presence (55.5%), while free play without additional equipment involved more stationary behavior and activities without equipment (46.9%). Conclusions: Although physical activity intensity did not differ across conditions, the structure, material context, and social organization of children’s activity varied, highlighting the practical importance of intentionally combining different outdoor activity formats to support diverse movement patterns in ECEC settings.

## 1. Introduction

Physical activity in early childhood plays a crucial role in children’s motor, cognitive, and socio-emotional development and provides a foundation for the adoption of long-term health-promoting behaviors. Given that preschool-aged children spend a substantial part of their day in early childhood education and care (ECEC) settings, the preschool environment is recognized as one of the key contexts shaping children’s everyday movement patterns [1,2]. Empirical evidence indicates that children’s physical activity levels vary considerably between preschools, reflecting the influence of organizational arrangements, the quality of the physical and material environment, and pedagogical approaches [3,4,5,6].

In efforts to increase children’s physical activity levels in ECEC settings, numerous interventions have been developed, primarily targeting changes in activity organization and adaptations of the educational environment. However, systematic reviews and meta-analyses suggest that the effects of such interventions are generally modest and inconsistent and largely dependent on contextual factors, as well as on the duration and design of the interventions [7,8]. An umbrella review by Lum et al. further emphasizes that, although many strategies show potential for increasing children’s physical activity, robust and consistent empirical evidence is still lacking for many of them, particularly under naturalistic, everyday preschool conditions [9].

Particular attention in the literature has been devoted to outdoor time, which is often viewed as an important opportunity for spontaneous movement and free play. Nevertheless, a systematic review and meta-analysis of studies conducted in preschool settings indicate that, despite greater opportunities for movement, children still spend a substantial proportion of outdoor time engaged in sedentary and low-intensity activities, while the proportion of moderate-to-vigorous physical activity (MVPA) is highly variable and often relatively low [10]. Consistent with these findings, available evidence suggests that even during outdoor time, children spend most of their time in light-intensity activities, whereas activities of moderate-to-vigorous intensity are comparatively infrequent [11,12]. At the same time, characteristics of the outdoor preschool environment may be associated with differences in overall physical activity levels, with equipment availability and spatial features shaping children’s behavioral patterns but not necessarily leading to substantial changes in activity intensity [13,14].

Within this context, some studies have focused on comparing different organizational formats of outdoor activities, most notably structured, educator-led physical activity and unstructured free play. Findings to date remain inconclusive, with some studies showing that overall physical activity intensity does not differ significantly between structured and unstructured conditions, at least during shorter play periods [15]. These findings highlight the importance of considering the contextual characteristics of children’s behavior during outdoor activities alongside quantitative assessments of activity intensity. Despite this, relatively few empirical studies have simultaneously examined both the intensity and the context of children’s physical activity when comparing different organizational forms of outdoor time. While previous research has predominantly focused on quantitative measures of activity intensity, less is known about how contextual characteristics of behavior, including activity type, equipment use, and social interaction, vary across conditions. This represents an important limitation, as children’s physical activity during outdoor play occurs within complex and dynamic social and environmental contexts.

Beyond differences in activity organization, the material characteristics of the environment may play an important role in shaping children’s behavior during outdoor play. In everyday ECEC practice, the availability of portable equipment, such as balls, hoops, or other movable objects, represents a common pedagogical variation. Such variations in the material environment can be interpreted as modifications of environmental affordances [16,17], shaping opportunities for engagement in different activity types, forms of social interaction, and patterns of equipment use.

However, empirical evidence remains limited regarding how variations in the material environment, independent of activity structure, relate to both physical activity intensity and the contextual characteristics of children’s behavior within naturalistic ECEC settings. This gap is particularly relevant in everyday practice, where differences in activity structure and material environment frequently co-occur and are rarely examined separately.

Direct observation methods provide a suitable approach for addressing this gap, as they enable the simultaneous assessment of physical activity intensity and contextual characteristics of behavior, including activity type, equipment use, and social context [18,19]. By capturing behavior as it occurs in real time, such approaches allow for a more comprehensive understanding of how physical activity unfolds within specific social and environmental contexts.

In line with the above, the aim of this study was to examine whether the intensity and context of preschool children’s physical activity differ across three outdoor conditions: structured, educator-led physical activity, outdoor free play, and outdoor free play with additional portable equipment. A further aim was to provide detailed contextual insight into patterns of children’s physical activity under naturalistic conditions using the OSRAC-P observational system.

Accordingly, the present study addressed the following research questions:

(1) Does physical activity intensity differ across structured activity, free play, and free play with portable equipment?
(2) Do contextual characteristics of physical activity (activity type, equipment use, and social context) differ across these conditions?

## 2. Materials and Methods

### 2.1. Participants and Setting

The study initially observed 120 preschool-aged children from two public early childhood education and care (ECEC) centers located in an urban area. The centers were selected based on the criterion of having broadly comparable spatial and material characteristics of their outdoor environments. Children were observed under three organizational physical activity conditions: structured, educator-led physical activity; outdoor free play; and outdoor free play with additional portable equipment. Due to the field-based design of the study, data were not available for all children across all conditions. Children with observations in only one condition (N = 36) were excluded from the analyses. The final analytical sample comprised 84 children with observations in at least two conditions, of whom 77 had complete data across all three conditions and were therefore included in the repeated-measures analyses of variance. Analyses of the distributions of activity types, equipment use, and social context were based on all available observations of children included in the analytical sample. The sample consisted of 42 boys and 42 girls, aged 4 to 7 years (M = 5.14; SD = 0.85). Observations were conducted during the morning hours in the children’s usual preschool environment.

### 2.2. Description of Activity Conditions

All observed activities were conducted within the same spatial and temporal framework of the preschool outdoor playground as part of the regular daily routine. Each of the three observed physical activity conditions lasted 30 min, ensuring temporal and contextual comparability, while differing in the degree of activity structure and the role of the educator.

Structured physical activity was implemented outdoors as a pre-planned, educator-led session under the direct guidance of the educator. The activity followed a clearly defined structure and sequence, consistent with typical practices of organized physical activity in early childhood education. It included introductory dynamic games aimed at activating the children, general preparatory exercises, an obstacle course, and rope-skipping tasks, followed by additional dynamic games. The session concluded with calming activities designed to gradually reduce movement intensity. Throughout the session, the educator played an active role in organizing, demonstrating, and regulating the flow of activities.

Outdoor free play took place without a predetermined structure or guided instructions, with children independently selecting activities, duration, and modes of engagement in play, using fixed playground equipment and materials regularly available in the preschool environment. The educator’s role was consistent with routine daily practice and was limited to supervising safety and occasionally joining play at the children’s request, without initiating or directing activities.

Outdoor free play with additional portable equipment was conducted under the same spatial and organizational conditions as free play without additional equipment. Children were provided with a range of portable equipment (e.g., frisbees, rackets, balls of various sizes, obstacles, balance elements, and skipping ropes) without prior explanation or demonstration of their use. Children independently and spontaneously chose whether and how to use the available equipment. The educator’s role was identical to that in the free play condition without additional equipment and was limited to safety supervision and reactive involvement in play only at the children’s request, without initiating or structuring activities. All provided equipment was simultaneously available to all children within the shared outdoor space, ensuring equal access throughout the activity period.

All three activity conditions were implemented on separate days within the regular preschool schedule. The conditions were applied in a fixed sequence across groups (structured activity, free play, and free play with portable equipment) and were conducted by the same educators for each group. The study was designed as a naturalistic field-based observation; therefore, conditions were not counterbalanced or randomized. Environmental factors such as weather conditions, playground surface, and group size were not experimentally controlled but reflected typical variations in everyday ECEC practice.

### 2.3. Instrument and Observation Procedure

The Observational System for Recording Physical Activity in Children, Preschool Version (OSRAC-P), was used to assess children’s physical activity. This validated direct observation system is designed for use in naturalistic preschool settings and enables the simultaneous recording of physical activity intensity and contextual characteristics of behavior, including activity type, equipment use, and social context [18].

Physical activity intensity was assessed using a five-level ordinal scale, where level 1 represents sedentary behavior and level 5 represents high-intensity activity. Activity type was coded according to OSRAC-P topographical categories, including stationary behavior, walking, running, jumping, climbing, throwing and catching, riding, and other activities. Equipment use was coded using predefined categories, including balls, climbing equipment, hoops, wheeled toys, sand play tools, sports equipment, and no equipment. The social context of activity was assessed using the OSRAC-P group composition category, distinguishing between solitary activity, one-to-one interaction with a peer or educator, and group activity with or without adult presence.

Observation was conducted using a momentary time sampling procedure. Each child was observed for 10 s, followed by 50 s for recording, resulting in one observation interval per minute. Each observation session lasted approximately 30 min per condition, yielding approximately 30 observation intervals per child per condition. This procedure represents an adaptation of the standard OSRAC-P protocol and was applied consistently throughout the study, allowing for the simultaneous recording of physical activity intensity and contextual characteristics of behavior in a naturalistic preschool environment.

In total, 7440 observation intervals were recorded across all participants and conditions. Due to minor variations in session duration and occasional non-codable intervals, the total number of valid observation intervals slightly differed from the theoretical maximum. Not all variables were coded in all intervals; therefore, the number of intervals included in specific analyses varied depending on data availability.

### 2.4. Observer Training and Interobserver Reliability

Observers were trained in the use of the OSRAC-P observational system [18] prior to data collection. Inter-observer agreement (IOA) was assessed during a pilot phase using point-by-point agreement based on 25 observation intervals of a single child coded concurrently and independently by five observers. Agreement values were 92.0% for physical activity intensity, 91.2% for activity type, 92.8% for equipment use, and 95.2% for social context, exceeding the recommended 90% criterion for all categories.

Following the establishment of satisfactory inter-observer reliability, each child was observed by a single observer during the main data collection.

### 2.5. Statistical Analysis

Primary analyses of differences between activity conditions were conducted using repeated-measures analysis of variance (ANOVA). These analyses were performed on participants’ mean observed physical activity intensity and on the mean proportions of time spent in each physical activity intensity category (sedentary, low, and moderate-to-high), calculated per child and per condition. The assumption of sphericity was tested using Mauchly’s test, which indicated no significant violation.

Differences in the distributions of activity types, equipment use, and the social context of activity (group composition) were examined using chi-square (χ^2^) tests of independence, with Cramer’s V calculated as a measure of effect size. For more detailed interpretation of significant χ^2^ results, standardized residuals were examined, with a threshold of |z| ≥ 2.0 considered indicative of meaningful deviations from expected frequencies. These analyses were used to describe patterns of contextual activity characteristics across conditions and were interpreted at the level of observation intervals rather than as independent effects at the individual child level.

To account for the hierarchical structure of the data and to examine the robustness of the findings, linear mixed-effects models with a random intercept for participants were additionally applied to analyze average physical activity intensity and the proportions of moderate-to-vigorous physical activity (MVPA) and sedentary behavior. The inclusion of a random intercept was justified through comparisons with a null model, with the intraclass correlation coefficient (ICC) indicating the proportion of variance attributable to between-child differences. Activity condition, child age, and sex were included as fixed effects, with activity condition specified as a within-subject factor. Model parameters were estimated using restricted maximum likelihood (REML).

An a priori power analysis was conducted using G*Power (version 3.1.9.7) [20] for a design with three repeated measurements (F tests; repeated-measures ANOVA, within-subject factors), assuming a significance level of α = 0.05 and statistical power of 1 − β = 0.80. A small effect size was assumed (f = 0.15), with a correlation among repeated measures of r = 0.50, resulting in a minimum required sample size of N = 73 participants. All analyses were performed using IBM SPSS Statistics (version 29.0).

## 3. Results

Descriptive statistics for physical activity intensity and the proportions of time spent in moderate-to-vigorous physical activity (MVPA), low-intensity activity, and sedentary behavior across conditions are presented in Table 1, Table 2, Table 3 and Table 4. Across all indicators, repeated-measures ANOVA revealed no statistically significant differences between activity conditions (all *p* > 0.05), with consistently small effect sizes.

Results from the linear mixed-effects models further indicated substantial variability in physical activity intensity between children, with approximately one-third of the total variance attributable to differences at the participant level (ICC ≈ 0.33). Activity condition did not emerge as a significant predictor of mean physical activity intensity after accounting for individual variability among children, F(2, 240) = 1.24, *p* = 0.292, while neither sex (*p* = 0.175) nor age (*p* = 0.622) was significantly associated with physical activity intensity. Fixed effects explained a small proportion of the variance in activity intensity (marginal R^2^ = 0.012), whereas the total explained variance was 34.2% (conditional R^2^ = 0.342).

Analyses of the proportion of MVPA likewise showed no significant effects of activity condition, sex, or age (all *p* > 0.05). In contrast, a significant association with age was observed for the proportion of sedentary behavior (B = 4.07, *p* = 0.016), indicating that older children spent a greater proportion of time engaged in sedentary activities, independent of activity condition and sex.

**Figure 1:**
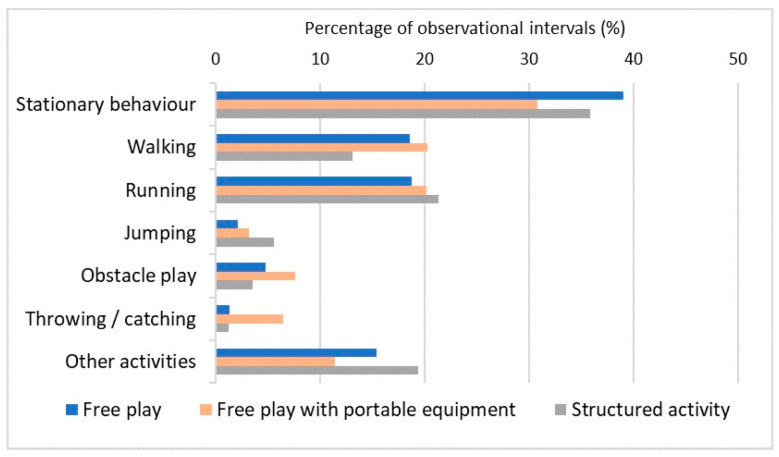
Percentage distribution of observed activity types during free play, free play with portable equipment and structured outdoor activity.

As shown in Table 5 and Figure 1, the distribution of observed activity types differed significantly across the three conditions. Visual inspection of Figure 1 indicates that free play without additional equipment was characterized by a higher proportion of stationary behavior, whereas free play with portable equipment involved a greater share of manipulative and locomotor activities. Structured activity showed a higher proportion of jumping and activities classified as “other”. Examination of standardized residuals (|z| ≥ 2.0) further indicated that free play with portable equipment was associated with higher-than-expected proportions of manipulative activities (throwing and catching), walking, and obstacle play, as well as a lower-than-expected proportion of stationary behavior. In contrast, free play without additional equipment was characterized by a higher-than-expected proportion of stationary behavior and lower-than-expected proportions of jumping and manipulative activities. Structured outdoor activity was associated with higher-than-expected proportions of jumping and activities classified as “other”, whereas manipulative activities and walking occurred less frequently than expected.

The distribution of equipment use differed significantly across the observed activity conditions (χ^2^(18, N = 4416) = 1572.98, *p* < 0.001, Cramer’s V = 0.422) (Table 6). Overall, free play without additional equipment was dominated by activities without equipment, whereas free play with portable equipment showed a broader and more frequent use of various equipment types. Structured activity was characterized by the predominant use of specific equipment, particularly hoops. Examination of standardized residuals (|z| ≥ 2.0) indicated that free play without additional equipment was characterized by a higher proportion of observation intervals with no equipment use and more frequent use of sand play tools, whereas other types of equipment occurred less frequently than expected. In contrast, free play with additional portable equipment was associated with higher proportions of ball use, sports equipment, climbing equipment, and wheeled toys, alongside a lower proportion of intervals without equipment. Structured physical activity was characterized by the predominant use of pre-specified equipment, particularly hoops, while balls, climbing equipment, sports equipment, and wheeled toys were used less frequently than expected.

The distribution of the social context of activity differed significantly across the observed conditions (χ^2^ (10, N = 7440) = 1522.54, *p* < 0.001, Cramer’s V = 0.29) (Table 7). Overall, free play conditions were characterized by a greater proportion of peer interactions and activities without adult involvement, whereas structured activity involved more adult presence and group-based activities. Examination of standardized residuals (|z| ≥ 2.0) indicated that free play was associated with higher proportions of solitary activities and activities conducted in groups without an adult, whereas one-to-one activities with an adult and group activities with an adult occurred less frequently than expected. Free play with portable equipment showed higher proportions of one-to-one interactions with peers and group activities without adult presence, while structured activity was associated with higher proportions of activities in groups with an adult and one-to-one interactions with an adult.

## 4. Discussion

The aim of this study was to examine whether the intensity and context of preschool children’s physical activity differed across three outdoor activity conditions: structured, educator-led physical activity; outdoor free play; and outdoor free play with additional portable equipment. The main findings indicate that children’s physical activity intensity did not differ significantly between the observed conditions, while clear differences were simultaneously identified in the contextual characteristics of activity, particularly in activity types, equipment use and social context. Although physical activity intensity did not vary across conditions, the differing activity forms and social frameworks were associated with distinct patterns of activity types, equipment use, and social organization during outdoor time.

### 4.1. Absence of Differences in Physical Activity Intensity

The absence of differences in physical activity intensity between structured activity, free play, and free play with additional portable equipment is consistent with a substantial body of previous empirical research conducted in ECEC settings. Using accelerometry to examine different forms of recess in a preschool context, Frank et al. found no significant differences in physical activity levels between structured and free-play conditions when analyses were conducted at the whole-sample level, despite pronounced individual variability among children [15]. Similarly, Tortella et al. reported no clear differences in physical activity levels between partially structured activity and outdoor free play, suggesting that changes in organizational format alone do not necessarily lead to changes in movement intensity [21].

Findings from the present study, derived from linear mixed-effects models, further corroborate this pattern. The analyses indicated that approximately one-third of the total variability in physical activity intensity (ICC ≈ 0.33) could be attributed to stable child-level differences, whereas the organizational condition of outdoor activity did not emerge as a significant predictor of physical activity intensity. These results suggest that differences in physical activity levels are more strongly related to children’s individual behavioral patterns than to differences in the organization of outdoor activities. This interpretation is consistent with previous research indicating that characteristics of the physical environment account for only a limited proportion of variability in preschool children’s physical activity [22].

Notably, the present study identified a positive association between child age and the proportion of sedentary behavior during outdoor time, with older children spending a greater proportion of time in sedentary activities irrespective of activity condition. This finding should be interpreted in light of previous research showing that age-related differences in sedentary behavior among preschool children are not unequivocal. A systematic review reported that child-level variables, including age, show no consistent or strong associations with sedentary behavior in ECEC settings [23], while empirical studies conducted in preschools have not identified significant differences in overall sedentary time between age groups during time spent in the institution [24,25]. These discrepancies may suggest that age-related patterns of sedentary behavior manifest selectively within specific activity contexts, such as outdoor time, and may depend on activity organization, measurement method, and the level at which behavior is analyzed.

In this context, previous research indicates that preschool children spend a substantial proportion of time in low-intensity activities, with moderate-to-vigorous physical activity occurring less frequently, regardless of activity organization [26,27,28].

The absence of higher levels of moderate-to-vigorous physical activity during structured physical activity may be related to organizational and pedagogical characteristics of this activity format. Although structured activities often include motorically demanding tasks, a substantial proportion of time may be devoted to task explanations, demonstrations, group organization, and waiting for turns, thereby reducing opportunities for sustained high-intensity movement. However, these factors were not directly measured in the present study and should therefore be interpreted with caution. In contrast, during free play, children have greater autonomy in selecting activities and regulating movement pace, which may be associated with shorter but more intense bouts of activity that, when averaged over time, approximate the intensity levels observed under structured conditions. These interpretations may help to contextualize the absence of differences in overall physical activity intensity across the observed outdoor activity conditions.

### 4.2. Context of Physical Activity in Outdoor Conditions

Although no differences in physical activity intensity were observed between the activity conditions, the results of this study indicate clear differences in the contextual characteristics of physical activity during outdoor time. Free play with additional portable equipment was characterized by a higher proportion of manipulative activities and more frequent equipment use, whereas structured physical activity involved a higher proportion of locomotor activities and more pronounced educator involvement in the activity itself. Free play without additional equipment more often included stationary activities and peer interactions, with minimal direct involvement of adults. These findings support previous research showing that environmental characteristics and equipment availability can shape patterns of children’s behavior, even when overall physical activity levels do not change. For example, Ng et al. demonstrated that modifications to preschool outdoor spaces may lead to changes in the types of activities children engage in, but not necessarily to increases in moderate-to-vigorous physical activity [13]. Similarly, Clevenger et al. emphasized that classifying physical activity by activity type provides different insights into children’s behavior than assessments based solely on location [29].

Observational studies further suggest that the availability of specific types of portable equipment may be associated with the nature and motor demands of activities, whereas other environmental elements, such as sand play, are more frequently linked to patterns of lower motor engagement [30]. In line with theoretical approaches emphasizing the concept of affordances, outdoor spaces in ECEC settings do not function as direct drivers of physical activity intensity, but rather as contexts that enable diverse forms of play and interaction with varying motor demands [17].

In addition to differences in activity type and equipment use, clear differences were also observed in the social context of physical activity. During free play, both with and without additional equipment, activities predominantly occurred without direct adult presence, most often in groups without an adult or in one-to-one interactions with peers [31]. The provision of portable equipment increased the frequency of peer interactions but did not result in greater adult involvement. In contrast, structured physical activity was characterized by substantially greater educator presence and group-based activities, with the adult assuming the role of organizer and regulator of the activity. Such social organization, however, does not necessarily lead to higher levels of physical activity, as structured frameworks may standardize movement pace and reduce individual variability in children’s behavior. These findings are consistent with theoretical perspectives that conceptualize the social dimension of the environment as an integral component of affordances shaping children’s play patterns, but not as a factor exerting a linear influence on physical activity intensity [16].

## 5. Strengths and Limitations

### 5.1. Methodological Implications

An important contribution of this study relates to the use of the OSRAC-P direct observation system, which enables the simultaneous examination of physical activity intensity and the contextual dimensions of children’s behavior under naturalistic, everyday preschool conditions. In contrast to studies that rely exclusively on accelerometry, an observational approach provides insight into the types of activities children engage in, the equipment they use, and the social contexts in which movement occurs [18,19]. This approach proved particularly valuable for interpreting findings in which overall physical activity intensity remained similar across conditions, while behavioral patterns and activity structures differed substantially.

The present findings further support the value of employing combined methodological approaches in research on physical activity in ECEC settings, especially when the aim is to understand not only the quantity but also the quality of children’s movement in naturalistic, everyday educational contexts.

### 5.2. Practical Implications

The results of this study indicate that decisions regarding the organization of outdoor time in preschools should not be guided solely by the goal of increasing physical activity intensity, but also by considerations of the context in which activity takes place. As different organizational conditions promote different activity types, patterns of equipment use, and forms of social interaction, the practical value of outdoor activities lies in their capacity to provide diverse patterns of observed movement behavior. The findings suggest that thoughtful variation and combination of different outdoor activity formats within daily or weekly routines may represent a simple and feasible strategy in preschool practice. For example, outdoor time could be organized by alternating short periods of structured, educator-led activities with periods of free play, or by introducing portable equipment during selected days of the week to encourage different types of engagement. In addition, periodically rotating or selectively introducing different types of portable equipment across sessions may be associated with greater variability in observed activity patterns and lower proportions of sedentary behavior, particularly among older children. Such an approach does not require extending outdoor time or introducing additional structural changes, but instead reflects a range of observed motor and social contexts within existing organizational frameworks. Moreover, the findings indicate that the role of the educator during outdoor time can be flexibly adapted to the goals of a given activity condition, with levels of guidance and child autonomy adjusted according to the type of activity. For instance, educators may take a more active role during structured activities, while adopting a supervisory and supportive role during free play or equipment-based activities. Such flexibility enables a balanced integration of structured and unstructured play elements, thereby contributing to greater variability in children’s movement patterns in everyday early childhood education and care practice. These implications are based on observed patterns and should be interpreted with caution regarding causal relationships.

### 5.3. Limitations and Future Research

Several limitations of this study should be considered when interpreting the findings. Observations were conducted over a limited data collection period and in a relatively small number of preschools (two ECEC centers). The specific physical characteristics of the outdoor environments (e.g., size, layout, and availability of fixed equipment) may also have influenced the affordances for movement, which may limit the generalizability of the results.

The exclusion of children with observations in only one condition may represent a potential source of selection bias, as differences between included and excluded participants were not systematically assessed. In addition, the possibility of a Hawthorne effect cannot be excluded, as children and educators were aware that observations were being conducted, which may have influenced behavior during outdoor activities.

Although analyses of physical activity intensity were performed on mean values per child and activity condition, these averages were derived from observation intervals that were hierarchically structured within children and centers. Future research could apply multilevel models directly at the level of observation intervals to more fully capture the temporal dynamics and variability of physical activity within specific conditions. Furthermore, the observational field-based design does not allow for causal inferences regarding activity conditions and children’s physical activity.

Inter-observer agreement was assessed on a relatively small number of observation intervals and based on a single child, which may limit the robustness of the reliability estimates. The use of extended observation intervals allowed for the simultaneous recording of physical activity intensity and contextual characteristics of behavior; however, this approach may have reduced sensitivity to detect very brief fluctuations in movement intensity. Future studies should therefore consider combining observational methods with objective measurement tools, such as accelerometry, as well as examining longer-term patterns of physical activity across different organizational outdoor activity conditions.

## 6. Conclusions

The findings of this study indicate that the overall intensity of preschool children’s physical activity does not differ significantly across different organizational forms of outdoor time, whereas the patterns and contexts in which this activity occurs differ markedly. Differences between conditions are primarily reflected in activity types, equipment use, and the social organization of movement, suggesting that structured activity, free play, and free play with additional portable equipment are associated with distinct patterns of activity and social organization. These findings underscore the importance of considering the contextual characteristics of physical activity, alongside intensity levels, when planning and interpreting outdoor activities in early childhood education and care settings.

## Figures and Tables

**Table 1:** Results of the repeated-measures ANOVA for mean physical activity intensity across three activity conditions.

Analysis	Value (M ± SD)
Structured activity	2.886 ± 0.652
Free play	2.818 ± 0.838
Free play with portable equipment	2.945 ± 0.819
Mauchly’s test of sphericity	W = 0.985, *p* = 0.573
Repeated-measures ANOVA (sphericity assumed)	F(2, 152) = 0.519, *p* = 0.596, partial η^2^ = 0.007
Linear trend	F(1, 76) = 0.267, *p* = 0.607
Quadratic trend	F(1, 76) = 0.823, *p* = 0.367

**Table 2:** Results of the repeated-measures ANOVA for MVPA (%).

Analysis	Value (M ± SD)
Structured activity	35.40 ± 18.35
Free play	31.59 ± 24.10
Free play with portable equipment	34.78 ± 24.85
Mauchly’s test of sphericity	W = 0.999, *p* = 0.957
Repeated-measures ANOVA (sphericity assumed)	F(2, 152) = 0.412, *p* = 0.663, partial η^2^ = 0.005

**Table 3:** Results of the repeated-measures ANOVA (RM-ANOVA) for low-intensity physical activity (LIGHT, %).

Analysis	Value (M ± SD)
Free play	37.14 ± 24.36
Free play with portable equipment	38.05 ± 25.26
Structured activity	37.58 ± 19.43
Mauchly’s test of sphericity	W = 0.983; *p* = 0.532
Repeated-measures ANOVA (sphericity assumed)	F(2, 152) = 0.033; *p* = 0.967; partial η^2^ < 0.001

**Table 4:** Repeated-measures ANOVA results for sedentary behavior (SED, %) across the three conditions.

Analysis	Value (M ± SD)
Structured activity	27.53 ± 20.32
Free play	31.08 ± 23.96
Free play with portable equipment	28.66 ± 24.06
Mauchly’s test of sphericity	W = 0.978; *p* = 0.436
Repeated-measures ANOVA (sphericity assumed)	F(2, 152) = 0.489; *p* = 0.614; partial η^2^ = 0.006

**Table 5:** Distribution of observation intervals by activity type and outdoor activity condition (OSRAC-P).

Activity Type	Free Play (n = 2460)	Free Play with Portable Equipment (n = 2610)	Structured Activity (n = 2370)
Stationary	960 (39.0%)	804 (30.8%)	852 (35.9%)
Walking	458 (18.6%)	531 (20.3%)	311 (13.1%)
Running	462 (18.8%)	526 (20.2%)	504 (21.3%)
Jumping	51 (2.1%)	84 (3.2%)	133 (5.6%)
Obstacle course	118 (4.8%)	198 (7.6%)	82 (3.5%)
Throwing/Catching	33 (1.3%)	170 (6.5%)	29 (1.2%)
Riding	39 (1.6%)	92 (3.5%)	0 (0.0%)
Other	339 (13.8%)	205 (7.9%)	459 (19.4%)

Note. The chi-square test of independence indicated a statistically significant association between activity condition and activity type, χ^2^ (14, N = 7440) = 513.09, *p* < 0.001, Cramer’s V = 0.186.

**Table 6:** Distribution of observation intervals by equipment use and outdoor activity condition (OSRAC-P).

Equipment	Free Play (n = 1348)	Free Play with Portable Equipment (n = 1826)	Structured Activity (n = 1242)
No equipment	580 (43.0%)	319 (17.5%)	446 (35.9%)
Balls	334 (24.8%)	629 (34.4%)	152 (12.2%)
Hoops	25 (1.9%)	55 (3.0%)	378 (30.4%)
Climbing equipment	94 (7.0%)	195 (10.7%)	28 (2.3%)
Sports equipment	6 (0.4%)	226 (12.4%)	7 (0.6%)
Wheeled toys	39 (2.9%)	106 (5.8%)	0 (0.0%)
Sand play tools	98 (7.3%)	16 (0.9%)	26 (2.1%)
Other	96 (7.1%)	199 (10.9%)	167 (13.4%)
Swing	44 (3.3%)	44 (2.4%)	28 (2.3%)
Slide	32 (2.4%)	37 (2.0%)	10 (0.8%)

Note. The chi-square test of independence indicated a statistically significant association between activity condition and type of equipment used, χ^2^ (18, N = 4416) = 1572.98, *p* < 0.001, Cramer’s V = 0.422. The analysis was conducted on a subset of observation intervals for which equipment use was coded.

**Table 7:** Distribution of observation intervals by group composition (social context) across outdoor activity conditions (OSRAC-P).

Group Composition	Free Play (n = 2460)	Free Play with Portable Equipment (n = 2610)	Structured Activity (n = 2370)
Solitary	194 (7.9%)	60 (2.3%)	89 (3.8%)
One-to-one with an educator	9 (0.4%)	11 (0.4%)	88 (3.7%)
One-to-one with a peer	662 (26.9%)	823 (31.5%)	392 (16.5%)
Group with an educator	411 (16.7%)	237 (9.1%)	1171 (49.4%)
Group without an adult	1154 (46.9%)	1449 (55.5%)	630 (26.6%)
Cannot tell	30 (1.2%)	30 (1.1%)	0 (0.0%)

Note. The chi-square test of independence indicated a statistically significant association between activity condition and group composition, χ^2^ (10, N = 7440) = 1522.54, *p* < 0.001, Cramer’s V = 0.29.

## Data Availability

The data presented in this study are not publicly available due to privacy and ethical restrictions.
